# Stable Au–C bonds to the substrate for fullerene-based nanostructures

**DOI:** 10.3762/bjnano.8.109

**Published:** 2017-05-17

**Authors:** Taras Chutora, Jesús Redondo, Bruno de la Torre, Martin Švec, Pavel Jelínek, Héctor Vázquez

**Affiliations:** 1 Institute of Physics, Academy of Sciences of the Czech Republic, Cukrovarnicka 10, Prague, Czech Republic; 2 Palacký University, RCPTM, Joint Laboratory of Optics, 17. listopadu 12, Olomouc, Czech Republic

**Keywords:** Au–C bonds, density functional theory (DFT), fullerenes, scanning tunneling microscopy (STM), sputtering

## Abstract

We report on the formation of fullerene-derived nanostructures on Au(111) at room temperature and under UHV conditions. After low-energy ion sputtering of fullerene films deposited on Au(111), bright spots appear at the herringbone corner sites when measured using a scanning tunneling microscope. These features are stable at room temperature against diffusion on the surface. We carry out DFT calculations of fullerene molecules having one missing carbon atom to simulate the vacancies in the molecules resulting from the sputtering process. These modified fullerenes have an adsorption energy on the Au(111) surface that is 1.6 eV higher than that of C_60_ molecules. This increased binding energy arises from the saturation by the Au surface of the bonds around the molecular vacancy defect. We therefore interpret the observed features as adsorbed fullerene-derived molecules with C vacancies. This provides a pathway for the formation of fullerene-based nanostructures on Au at room temperature.

## Introduction

In single-molecule electronics, the active element in an electronic circuit is a small molecule connected to two nanoelectrodes, and molecular chemical properties determine the characteristics of current flow. The reliable preparation and characterization of such nanostructures has been made possible by state-of-the-art scanning probe methods with which individual atoms and molecules can be manipulated. In parallel, the use of atomistic simulations, mainly based on density functional theory (DFT), has allowed for a detailed understanding of the basic mechanisms that determine the electronic and nanoscale transport properties [1]. For spintronics, small organic molecules are appealing since they feature weak spin–orbit interaction and long spin lifetimes [2–3].

The large pool of organic molecules opens the possibility of almost unlimited functionalities given the right molecular design [4]. Fullerenes are particularly well-studied molecules. Since their discovery in 1985 [5], fullerenes have played an important role in molecular surface science, organic photovoltaics and single-molecule electronics. Fullerenes can be deposited on a series of metallic and semiconducting substrates [6–8]. In molecular transport, they have been used both as target molecules as well as anchoring groups [9–12]. They have featured in spin transport studies, where spin currents can be achieved by encapsulating magnetic atoms or impurities inside the fullerene cage [13–18]. The adsorption of C_60_ on the metal surface determines the strength and spread of electronic coupling and conductance values [9–12]. For an archetypal electrode material in single molecule transport studies such as Au, however, their high mobility at room temperature can lead to a large spread in conductance or to problems in trapping the molecule at the interface [19–20]. It might therefore be desirable to achieve strong metal–molecule bonds that result are electronically transparent or exhibit a well-defined conductance. Au–C metal–molecule bonds were found to be highly conducting [21–22].

Here we report on the formation of stable fullerene-based nanostructures on Au(111) at room temperature in ultra-high vacuum (UHV) environment. These structures were realized by soft sputtering of fullerene films on the surface with Ar^+^ ions and were studied using scanning tunneling microscopy (STM). After sputtering, bright spots on the herringbone corners are observed, which we show to be adsorbed fullerenes with defects created by the sputtering process. The sputtering process is expected to result in the formation of vacancies in the fullerene molecules, where C atoms are knocked out. A series of fullerene fragments can be formed in the collision with high-energy atoms and ions. In our work, we gradually increased the energy of the incident ions starting from a low value until changes in the film morphology (in particular the spots on the herringbone elbows) were observed. We therefore hypothesize that the damaged fullerenes in our study are C_59_ molecules, an assumption discussed below. C_59_ molecules have the highest energetic stability (difference between the cluster energy and the sum of the energy of the individual C atoms) after C_60_ [23–25]. These findings are corroborated by total-energy DFT simulations.

Since the diffusion of fullerenes on Au is very fast at room temperature, individual molecules cannot be stabilized and contacted outside islands. This has important consequences for single-molecule transport, where it would be desirable to have reliable and stable metal–molecule contacts. In the case of molecular spintronics, the stable fullerene-based structures proposed here might be useful for transport studies on magnetic atoms and impurities encapsulated inside molecules based on fullerenes.

## Results and Discussion

### STM room-temperature measurements

**Figure 1:**
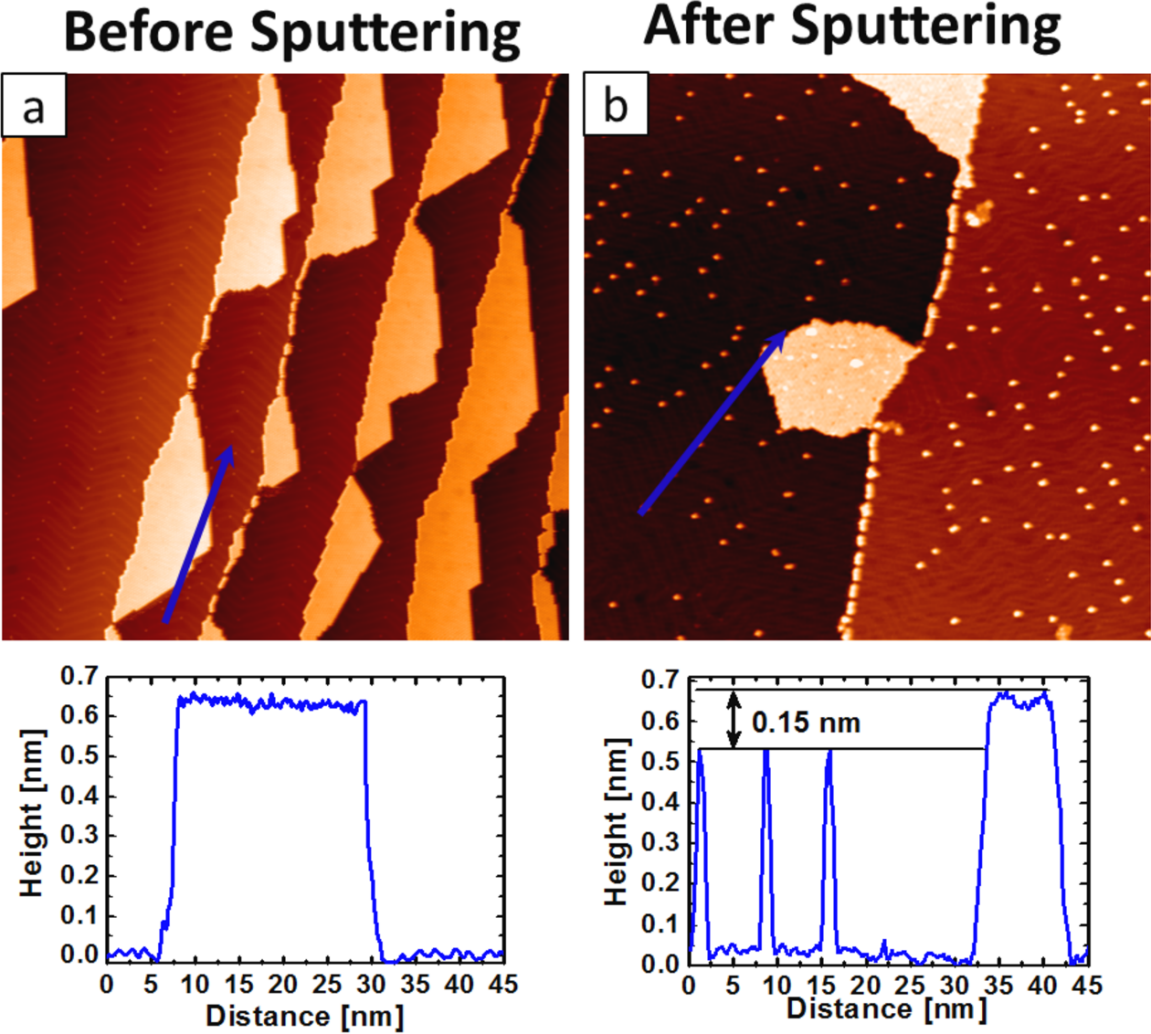
(a) (200 × 200 nm^2^) High-resolution STM image (*U*_b_ = −0.5 V, *I*_s_ = 0.3 nA) of Au (111) after deposition of C_60_. The molecules formed self-assembled islands, attached to the surface-terrace edges as expected. (b) (200 × 200 nm^2^) High-resolution STM image (*U*_b_ = −0.6 V, *I*_s_ = 0.3 nA) of the system after sputtering with 120 eV Ar^+^ ions for 5 min. Single molecules were detached from the islands as the result of the sputtering process. Profiles measured along the indicated blue arrows reveal the apparent height differences between isolated molecules and those inside the island.

Figure 1a shows a constant-current STM image acquired at room temperature after the deposition of C_60_ molecules on the reconstructed Au(111) surface [26–28]. The deposition process was performed at room temperature. In this initial state of adsorption, we observe that almost all the C_60_ molecules are adsorbed at the terrace edges of the monoatomic steps. Molecules are assembled into one-dimensional islands or short chains along the steps. This can be attributed to the increased local reactivity of the step edges [29–31]. STM images taken after Ar^+^ bombardment (120 eV, 5 min) [32–34] of the system (Figure 1b) show single bright dots on the surface, which correspond to individual molecules disjoined from islands as a result of the sputtering process. Line profiles (indicated by blue lines in Figure 1b) reveal an apparent height difference of approximately 0.15 nm between the individual molecules and those inside the island.

**Figure 2:**
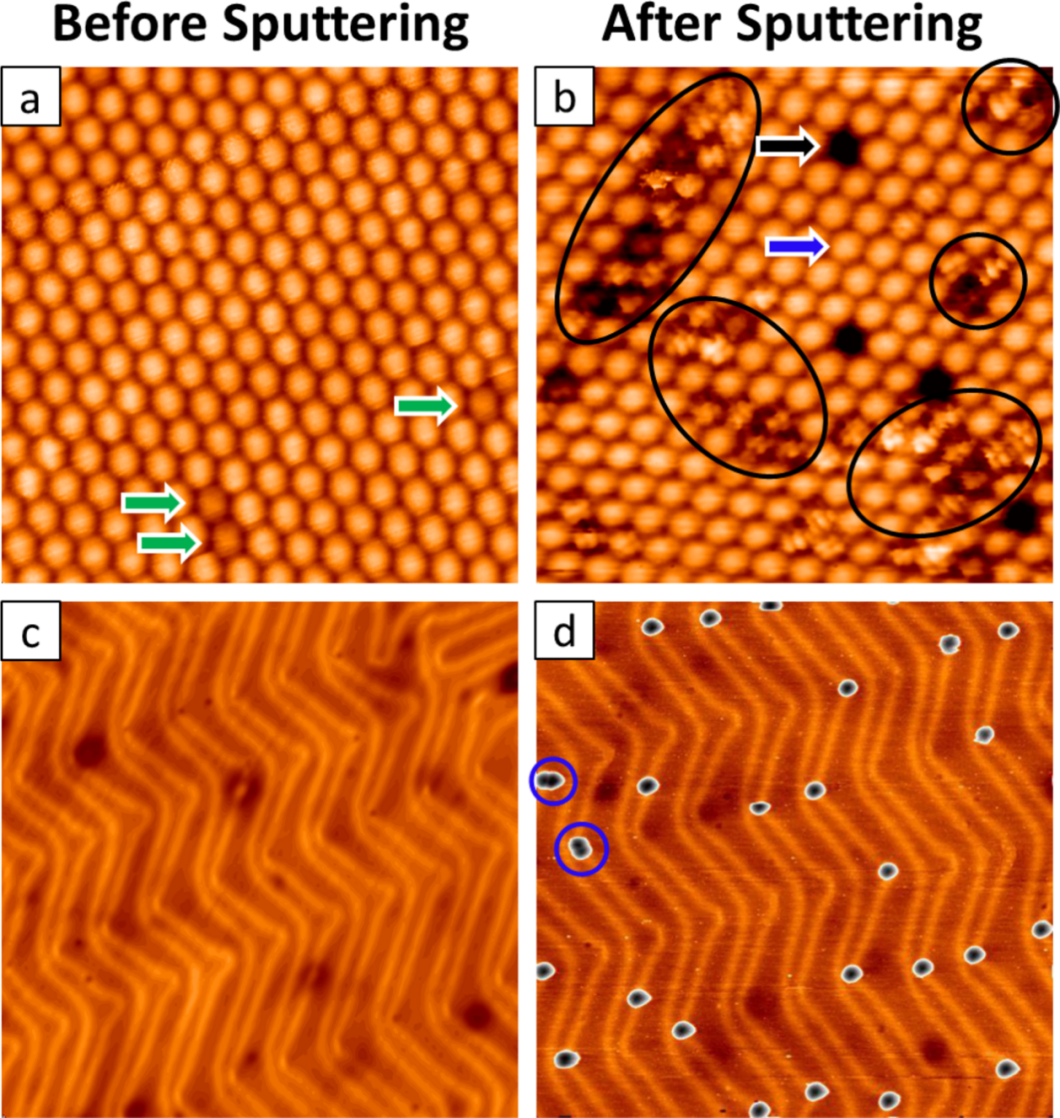
(a) (14 × 14 nm^2^) STM image (*U*_b_ = −2.5 V, *I*_s_ = 0.3 nA) of a fullerene island before Ar^+^ bombardment. (b) (14 × 14 nm^2^) STM image (*U*_b_ = 0.6 V, *I*_s_ = 0.3 nA) of the fullerene island after Ar^+^ bombardment. The blue arrow indicates a pristine C_60_ molecule. The black arrow points to a vacancy inside the island. Black circles mark regions with modified molecules. (c) (65 × 65 nm^2^) STM image (*U*_b_ = 1.8 V, *I*_s_ = 0.3 nA) of the herringbone reconstruction before Ar^+^ bombardment. (d) (65 × 65 nm^2^) STM image (*U*_b_ = 0.5 V, *I*_s_ = 0.09 nA) of single molecules attached to the herringbone elbow sites. Two dimers are enclosed by blue circles.

Figure 2a shows a high-resolution STM image of the close-packed arrangement of C_60_ inside the island after deposition [35–37]. In addition, we observe dim molecules (indicated by green arrows), which can be attributed to C_60_ molecules above gold vacancies [29,37]. Closer inspection of molecules inside the island after Ar^+^ ion bombardment (Figure 2b) enables the sorting of the molecules in the island according to their appearance. We can easily identify pristine C_60_ (blue arrow). Also, we observe regions in the island (indicated by black circles) corresponding to modified molecules, with a variation in the topographic heights. We assume that the varying apparent heights of these molecules inside the island stem from different adsorption geometries and possibly the local influence of neighboring molecules. Finally, we observe dark spots in the islands (black arrows in Figure 2b), which we can attribute to holes formed due to the ion bombardment and subsequent departure of the fullerenes from the islands.

Figure 2c shows a high-resolution STM image of reconstructed Au(111) after C_60_ deposition prior to the Ar^+^ ion bombardment. From this image, it is clear that no molecules are seen at the elbow sites before soft sputtering. In Figure 2d we observe the adsorption pattern of isolated molecules that were disjoined from the islands after sputtering. Importantly, these molecules bind to the elbow sites of the herringbone reconstruction of the substrate. This can be explained by the increased reactivity of the elbow sites, so-called Shockley partial dislocations of the Au bulk [38–39]. The faulty structure of the elbow site makes it a favorable nucleation site for the functionalized molecules to bind. STM images also show the presence of dimer structures bound at the elbow sites (indicated by blue circles). We attribute these features to be dimers of molecules damaged during the sputtering. The number of observed dimers was very limited.

We turn to the features on the herringbone corners. Given the higher reactivity of these elbow sites, we consider the possibility of the bright spots being normal C_60_ molecules and for the structures on the herringbone corners being unrelated to the formation of molecular defects. However, this scenario can be ruled out since these spots are only observed after sputtering of the fullerene film. First, without sputtering, C_60_ molecules are highly mobile on terraces at room temperature and form islands that are adsorbed at step edges. Second, the creation of reactive sites in the Au surface due to sputtering and to which normal C_60_ molecules could bind, can also be excluded: no features on the elbow sites were observed when sputtering the clean Au surface prior to C_60_ deposition. Molecules bound to the herringbone corner sites were only observed after soft sputtering of the fullerene films, implying that these adsorbates result from an increased reactivity of the molecules after sputtering.

### Isolated fullerenes with C vacancies

In order to understand the STM measurements, we carried out electronic-structure calculations based on DFT, focusing on fullerene molecules with vacancy defects where the missing C atoms result in increased reactivity and stronger binding with the substrate. We consider C_59_ molecules, resulting from the removal of a single C atom. While high-energy collisions can result in a wide range of products after removal of a series of fragments [23], it has been shown that sputtering of carbon materials with such low energies as in our case results in predominantly single vacancies [32–34]. C_59_ molecules also have the highest energetic stability after C_60_ [24–25] and, as described below, result in strong and stable bonds to the Au(111) surface, in particular stronger than those of C_58_. We therefore study the binding of C_59_ species to the substrate which, as detailed below, explains the STM observations.

**Figure 3:**
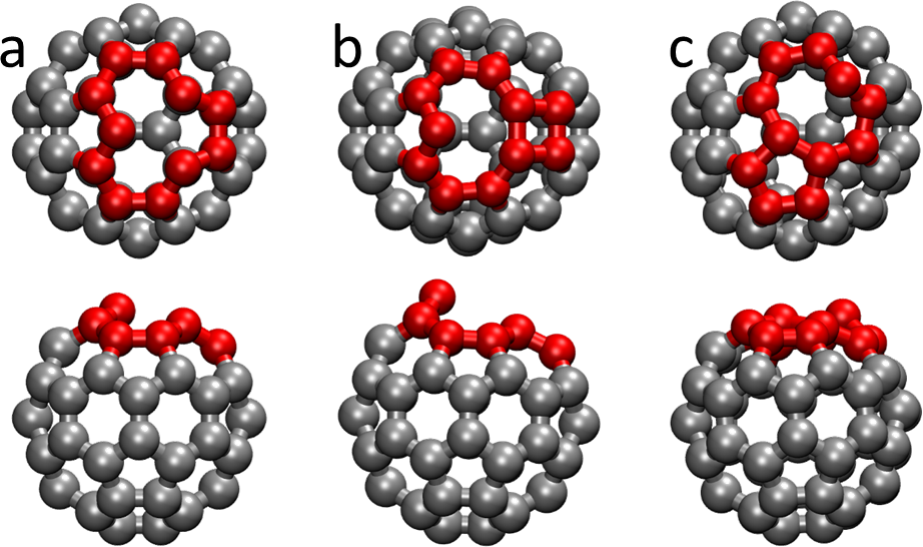
Top and side views of the computed structure of C_59_ structural isomers. Carbon atoms around the vacancy are shown in red. (a) Initial structure for geometry optimization, obtained from a C_60_ molecule by removing one atom. (b) Isomer where the atoms around the vacancy form one four- and one nine-membered ring. (c) More stable isomer having eight- and five-membered rings.

We start by discussing the structure of isolated C_59_ molecules. From the equilibrium C_60_ molecule, we remove a C atom and explore low-energy structures by optimizing the geometry. The removal of an atom from the C_60_ molecule results in many unsaturated bonds that induce a geometric rearrangement of the molecule. In the calculations, we find two different structural isomers (Figure 3), depending on how the fullerene vacancy is healed. In the first isomer, the atoms surrounding the vacancy rearrange to form two rings, one consisting of four atoms, and the other of nine atoms. In the other one, the C atoms around the vacancy assemble into a ring of eight atoms, and another ring of five atoms. Notice that in both structural isomers two C atoms belong to both rings, but for clarity we choose to name them according to the total number of atoms in each ring. At the optimized structures, both C_59_ isomers have a carbon atom protruding from the shape of a C_60_ molecule or that of a C_60_ molecule with one missing C atom (the starting geometry of the structural optimizations). Despite the structural rearrangement, interatomic C–C bond distances are not dramatically altered. Calculated interatomic bond distances for C_60_ are 1.42 and 1.47 Å, to be compared to the reported values of 1.40 and 1.46 Å [40–41]. For the 4,9- isomer, C–C distances around the vacancy are in the range of 1.42–1.49 Å, while for the 8,5-isomer the calculated values are between 1.40 and 1.51 Å. When comparing the total energy of both species we find the isomer with 8- and 5-atom rings to be more stable by ca. 0.9 eV than the 4,9-isomer, consistent with previous quantum chemical calculations [25]. Therefore, when considering the adsorbed defected fullerenes on the surface we study the 8,5-isomer only.

### Fullerenes with defects adsorbed on the Au(111) surface

**Figure 4:**
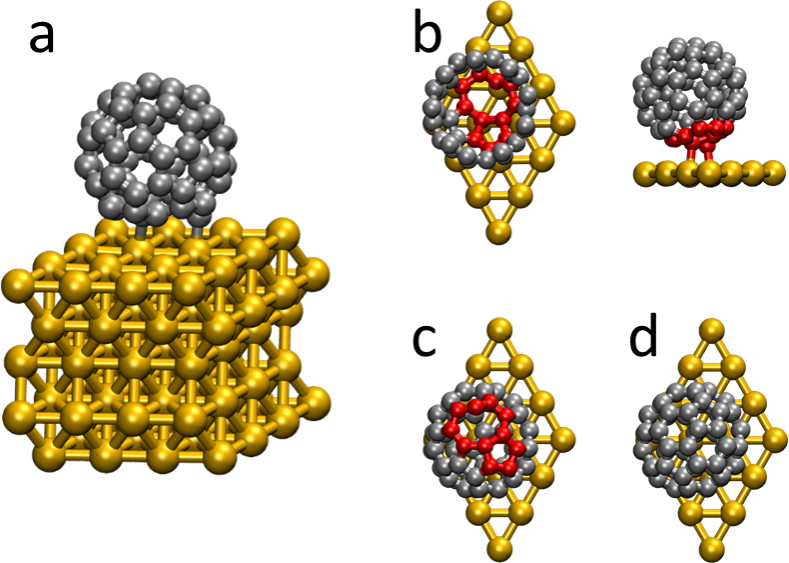
(a) Unit cell used in the calculations. (b) Top and side views of the 8,5-isomer with the single vacancy close to the metal surface. In the top view, the upper C atoms have been removed for clarity. (c) Top view of the 8,5-isomer with the vacancy away from the surface. (d) Fullerene with no defects (C_60_). Carbon atoms around the vacancy are shown in red.

We now describe the adsorption of this 8,5-fullerene with vacancy defect on the (111) surface of Au using DFT simulations. The herringbone reconstruction arises from the 22×√3 reconstruction of the Au(111) surface. However, the calculation of the very large supercells needed to explicitly describe this reconstruction would require a huge computational effort [42]. We therefore follow previous works and study the adsorption on the ideal (111) surface. Figure 4a shows the unit cell used in the calculations, illustrated for pristine fullerene (C_60_). There are five Au layers, each consisting of 16 atoms. We calculate the fullerene molecule before and after sputtering, where we model it as having 60 and 59 atoms, respectively. In both cases, above the molecule there is a large vacuum gap. Technical details of the calculations are given in the Experimental section at the end of the paper.

We first screen the possible adsorption geometries by carrying out structural optimizations starting from a series of initial metal–molecule structures. We investigated several initial geometries where the C_59_ molecule was rotated or its center of mass had been shifted, in order to explore the metal–molecule interaction. We considered geometries where the fullerene vacancy was oriented towards the Au substrate (“defect-down” structure) as well as towards the vacuum (“defect-up” structure). Figure 4b–d show the optimized geometries for the fullerene molecules with vacancy defect having the vacancy towards the interface or towards the vacuum, and for the adsorbed pristine C_60_ molecule. We find that, upon adsorption, the calculated interatomic C–C bond distances are only slightly changed compared to the isolated 8,5-isomer. When the vacancy is oriented towards the vacuum, the changes in the C–C bond distances are negligible. In the defect-down geometry they are larger, as expected, with the smaller five-atom ring exhibiting smaller bond distance variations upon adsorption (mean change less than 0.005 Å) than the eight-atom ring (mean change of ca. 0.025 Å). This is consistent with the intuitive idea that the eight-atom ring is more reactive, and in fact is found to be closer to the metal surface in the optimized geometry. From the calculations, the binding energy of the defect-down geometry (Figure 4b) is ca. 1.6 eV. This is much higher than that of the defect-up (Figure 4c) and the pristine C_60_ (Figure 4d) structures. The calculated binding energies of these two structures is (in the absence of van der Waals forces) close to zero. This indicates that changes in the electronic structure arising from the vacancy when it is oriented towards vacuum do not significantly affect the metal–molecule contact. In contrast to the value of the defect-down structure, previous calculations on C_60_/Au(111) have established that the binding energy results almost solely from van der Waals interactions [43]. Finally, we also considered the case of a double vacancy. Calculations for the binding of a C_58_ molecule with the defect pointing towards the metal result in a binding energy close to 0.6 eV. This value is significantly smaller than that of C_59_, further supporting the idea that the bright spots observed in STM are fullerenes with single vacancies.

The optimized geometry of the defect-down C_59_ structure has three different C atoms separated by less than 2.5 Å from an Au atom in the surface layer. As expected, one of these atoms is the C atom protruding from the eight-atom ring, consistent with the intuitive notion of its high reactivity and readiness to form bonds with the substrate. As seen in Figure 4b, these small Au–C distances result from the three C atoms being close to an atop position with respect to the Au layer. For comparison, these values are slightly larger than the Au–C distances of ca. 2.1 Å found in other molecular nanostructures [21–22]. In our simulations, we found another local minimum of the C_59_ defect-down configuration with a smaller binding energy of 1.2 eV. In this geometry, the protruding C atom in the eight-atom ring is also close to a surface Au atom, while other C atoms in the eight- and five-membered rings are further away. This shows that, although several atoms around the fullerene vacancy contribute to the binding energy at the interface, the most important contribution comes from the apical C atom sticking out of the former icosahedral structure. In the case of the defect-up and the pristine structures (Figure 4c,d), a C–C bond shared by two hexagons relaxed to a position above a Au surface atom. This was previously found to be a favorable binding site for C_60_/Au(111) [37]. Changes in the orientation of the defect-up structure resulted in minor variations in the calculated binding energy [43]. This is again consistent with the notion that when the vacancy is oriented towards vacuum, the carbon atoms close to the Au surface are relatively unaffected and the binding to the metal is similar to that of pristine C_60_.

To sum up, the passivation of the bonds of the C atoms around the vacancy defect by the Au surface results in the formation of metal–molecule bonds and an energy gain of 1.6 eV. On the other hand, a vacancy exposed towards the vacuum would by very unstable and energetically unfavorable and it is unlikely that it would be present in experiment. Finally, from the calculations, the height of the defect-down fullerene is ca. 0.9 Å lower than that of the pristine C_60_ molecule.

**Figure 5:**
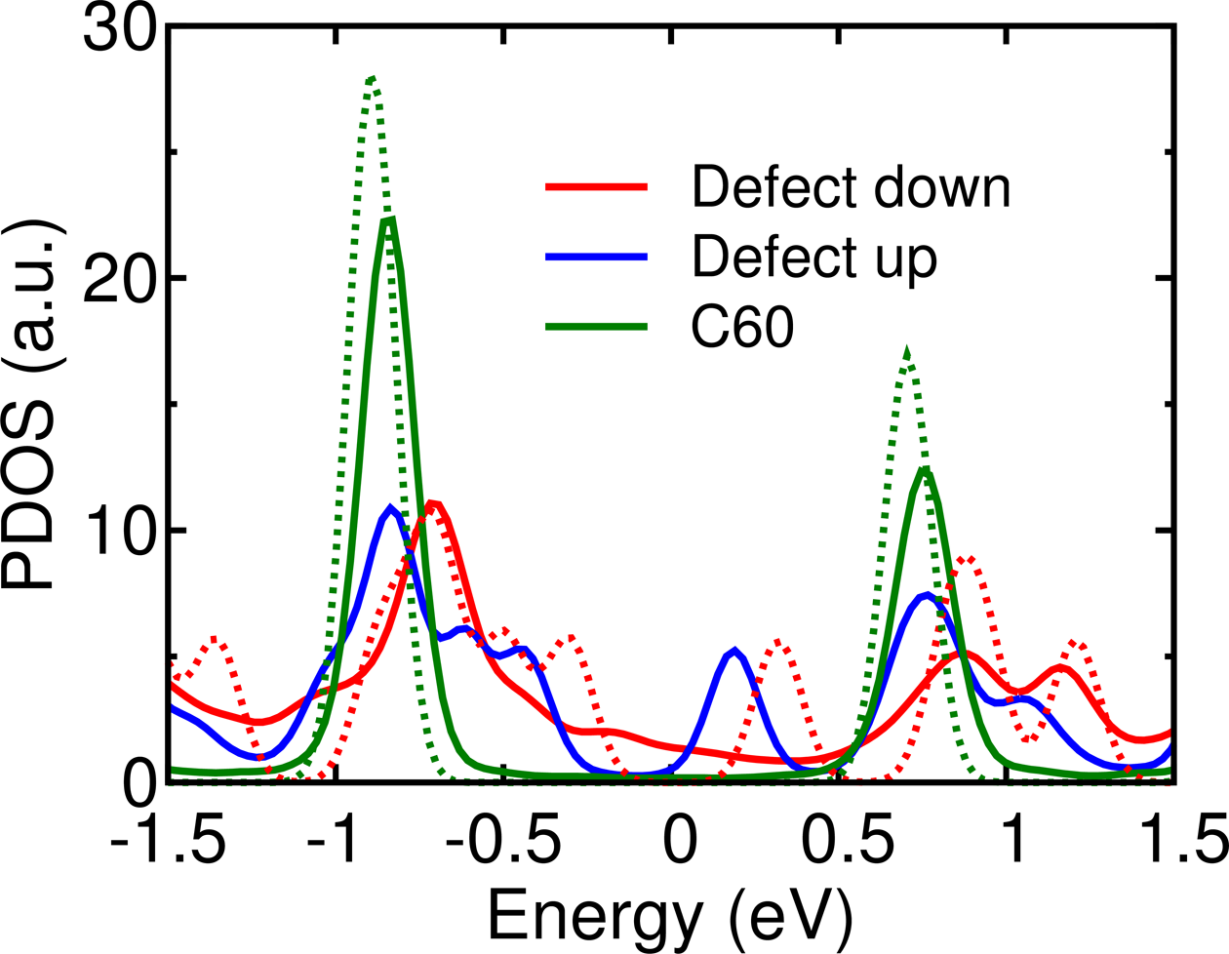
Calculated DOS of fullerenes with and without vacancy defects adsorbed on Au(111). In the case of defects, the molecule was adsorbed with the vacancy close to the surface (“defect-down”) or towards the vacuum layer (“defect-up”). The DOS of isolated molecules are shown as dashed lines.

Finally, we turn to the electronic properties of the adsorbed fullerenes with vacancy defect, and compare them to those of the C_60_/Au(111) system. Figure 5 shows the calculated density of states (DOS) of the isolated molecule (dashed lines) and junction (solid lines) projected onto the molecular atoms. Upon adsorption, the calculated spectrum of C_60_ is not appreciably modified, nor is that of C_59_ for the defect-up geometry. When the defect is adsorbed facing the substrate, however, significant changes are seen with respect to the isolated molecule. Spectral features, especially in the empty part of the spectrum, are broadened due to hybridization with metal states. Three scenarios at the interface are compared: the C_59_ molecules adsorbed with the vacancy towards the Au substrate or away from it, and the case of C_60_ for comparison. The spectrum of C_60_ on Au is well known [29,44–46]. Fullerene has a three-fold degenerate LUMO and a five-fold degenerate HOMO. In Figure 5 these are the peaks at about 0.8 and about −0.9 eV. The vacancy defect in the fullerene is related to the existence of states in the former gap of the molecule. For the defect-up geometry this is clearly seen in the peaks at 0.2 and around −0.5 eV, which can be explained by the breaking of degeneracy of one empty and two occupied states. Other molecular states are relatively unaffected compared to C_60_/Au(111). When the vacancy is adsorbed towards the substrate, the Au–C bonds result in the broadening of the molecular spectrum, and there are broad features in the former energy gap. Identifying individual peaks and comparing them with C_60_ is more difficult but the occupied part of the spectrum seems to have changed more than the empty states upon adsorption. Unfortunately, attempts to reliably measure at room temperature the d*I*/d*V* spectrum of molecules adsorbed at the herringbone elbow sites or in islands were unsuccessful.

## Conclusion

To summarize, we presented a combined theoretical–experimental study of sputtered fullerene-based films on Au(111). We carried out STM measurements at room temperature in UHV. Initially we observed C_60_ molecules forming islands or chains at terrace edges of monoatomic Au steps. After soft sputtering, bright spots were visible at the Au herringbone corners. Line scans revealed these spots to have an apparent height difference of 1.5 Å with respect to fullerenes in islands. We interpret these bright spots as fullerene molecules with vacancies created by the sputtering process. DFT-based calculations show that C_59_ fullerenes with single defects are consistent with experimental findings. The vacancy created by the removal of a C atom from a fullerene molecule results in structural rearrangement and increased molecular reactivity. We showed that C_59_ molecules adsorbed with the defect close to the surface have a binding energy on Au that is 1.6 eV higher than that of C_60_. This results from the passivation of C unsaturated bonds around the defect by the Au surface atoms. The calculated metal–molecule structure has several Au–C bond distances below 2.5 Å at the interface. This favorable binding configuration of the fullerene defect is consistent with the stable isolated molecules observed experimentally at the herringbone corners after sputtering. Our work thus provides a pathway for the formation of strong metal–molecule anchors for fullerene-based nanostructures at room temperature.

## Experimental

### Deposition and sputtering of C_60_

Experiments were performed in ultrahigh vacuum, variable temperature STM (VT-STM), with base pressure below 5 × 10^−10^ mbar. Typically, six cycles of Ar^+^ ion sputtering (1 kV, 10 min) and annealing (600 °C, 5 min) were required to obtain samples with overall cleanliness suitable for achieving the atomic resolution by means of STM. For deposition, we employed a custom-made thermal evaporation source, which contained a pocket made of tantalum, suitable for the evaporation of molecules such as C_60_. During the deposition, the evaporation source and substrate were placed inside a vacuum chamber with a base pressure around 5 × 10^−10^ mbar. Before every deposition, the C_60_ source was preheated to 360 °C and degassed for 5 min to remove contaminations. After this procedure the sample was transferred into the STM head for the deposition of C_60_ by heating the evaporation source at 420 °C. In order to remove C atoms from the C_60_ molecules, the sample was bombarded with Ar^+^ ions (120 eV, 5 min).

### DFT-based calculations

We use the DFT code Siesta [47] for the calculation of the adsorption and electronic properties. We used single-zeta polarized orbitals for gold and a double-zeta polarized basis for carbon atoms. Exchange–correlation was described with the Perdew–Burke–Ernzerhof implementation of the Generalized Gradient Approximation (GGA) [48]. Each Au layer consisted of 16 atoms and five layers were used in the calculations. A vacuum gap of about 10 Å was introduced above the topmost molecular atom to avoid interaction with the cell images in the *z*-direction. Interface geometries were optimized using the Conjugated Gradient algorithm. We used a 2 × 2 Monkhorst–Pack grid for the *k*-point sampling of the Brillouin zone. The position of Au atoms in the surface layer and C atoms was relaxed until the forces acting on these atoms were smaller than 0.02 eV/Å. Projected DOS curves were calculated using a denser 15 × 15 Monkhorst–Pack *k*-point grid at optimized geometries. For the calculation of fullerene binding energies, ghost orbitals were used to correct for basis set superposition errors [49].
